# Association of Plasma Homocysteine with Self-Reported Sleep Apnea Is Confounded by Age: Results from the National Health and Nutrition Examination Survey 2005-2006

**DOI:** 10.1155/2012/634920

**Published:** 2011-12-29

**Authors:** Tushar P. Thakre, Manju Mamtani, Shweta Ujaoney, Hemant Kulkarni

**Affiliations:** ^1^Lata Medical Research Foundation, Nagpur, 440022, India; ^2^Cleveland Clinic Sleep Disorders Center, Cleveland, OH 44195, USA; ^3^Advanced Education in General Dentistry program, Case School of Dental Medicine, Cleveland, OH 44206, USA; ^4^Texas Biomedical Research Institute, San Antonio, TX 78227-5301, USA

## Abstract

High levels of plasma homocysteine are implicated in the pathogenesis of cardiovascular diseases especially if accompanied by sleep apnea, but a direct pathogenetic link between plasma homocysteine levels and obstructive sleep apnea is debatable. This association can have far-reaching public health implications considering the inverse association between folate and plasma homocysteine. We used data from the 2005-2006 cycle of the National Health and Nutrition Examination Survey (NHANES) to test the hypothesized associations. Of the 4490 subjects included in analysis, 177 reported sleep apnea. Age-standardized and design-effect-corrected prevalence rates were differential across gender, plasma homocysteine, and red cell folate status. Plasma homocysteine was positively correlated with age (*r* = 0.38, *P* < 0.0001). Multivariate analyses using sociodemographic and clinical covariates demonstrated that plasma homocysteine levels retained their respective associations with self-reported sleep apnea in all models except when age was included as a covariate. Our results demonstrate that the claimed association of plasma homocysteine with sleep apnea may be confounded by age.

## 1. Introduction

Obstructive sleep apnea (OSA)—a disorder in which a person frequently stops breathing during sleep—results from an obstruction of the upper airway that occurs because of inadequate motor tone of the tongue and/or airway dilator muscles. In the United States, the prevalence of OSA is estimated to be 3–7% in men and 2–5% in women [1]. In addition, up to 93% of women and 82% of men may already have an undiagnosed moderate to severe OSA [2]. Further, the comorbid occurrence of OSA with obesity is well-recognized: prevalence of OSA is reported to be 41% among patients with a body mass index (BMI) greater than 28 Kg/m^2^ and as high as 78% in morbidly obese patients who present for bariatric surgery [3, 4]. Of greater interest and importance, however, is the association of OSA with cardiovascular disorders [5]. OSA has been identified as a crucial intermediate factor in the pathophysiology of hypertension, ischemic heart disease, arrhythmias, stroke and diabetes. It has been shown that habitual snorers are at a 2 times higher likelihood of developing type 2 diabetes independently of other covariates [6]. Also, treatment of sleep-disordered breathing is known to improve outcomes after congestive heart failure and stroke [7, 8].

A possible mechanism for the strong correlation between OSA and cardiovascular risk factors is the concomitant association of plasma homocysteine levels with both these disorders [9–11]. Homocysteine—a homologue of cysteine is a biosynthesized amino acid in the metabolism of methionine. Its production correlates with the deficiency of vitamins B6, B12, and folic acid. Indeed, plasma homocysteine levels are considered a good indicator of the deficiency of these vitamins [12]. The importance of homocysteine metabolism in the initiation or precipitation of cardiovascular diseases can be appreciated by the fact that the attributable risk of hyperhomocysteinemia in the epidemiology of cardiovascular diseases is nearly 25% and competes with that of other well-known factors like smoking and hyperlipidemia [13]. In contrast, the association of plasma homocysteine levels with the risk, severity and long-term complications of OSA is less clear. Over the last decade, 15 epidemiological studies [14–28] have examined the potential association of plasma homocysteine levels with OSA under varying scenarios (Table 1). Of these, nine studies [4–6, 12, 16, 17, 21, 24, 26] have reported overall or subgroup-specific association while six studies [3, 8, 9, 13, 20, 23] have not found any association. Some elegant reviews [9, 10] in this area have also not been fully conclusive about such an association.

In concept, this association—if statistically and truly existent–proffers an enticing opportunity for simple public health measures like vitamin supplementation for the prevention and control of OSA as well as its cardiovascular implications [29]. However, as can be gleaned from Table 1, most of the studies in this area have been based on relatively small sample sizes which somewhat limits the public health implication of these results. We therefore analyzed a large nationally representative sample which was collected during the National Health and Nutrition Examination Survey (NHANES) in the 2005-2006 cycle. Here we report the results of our investigation into the association of plasma homocysteine, and folate levels in the plasma as well as the red blood cell (RBC) with the risk of self-reported sleep apnea in an epidemiological context.

## 2. Methods

### 2.1. The NHANES 2005-2006 Dataset

The National Health and Nutrition Examination Survey (NHANES) is an annual survey conducted by the National Center of Health Statistics (NCHS) of the Centers for Disease Control and Prevention (CDC), Atlanta, Georgia, USA. It comprises a combination of interviews, physical examination and laboratory tests to assess the health and nutritional status of adults and children in the United States. The NHANES 2005-2006 and 2007-2008 cycles contain a questionnaire to identify subjects with diagnosed sleep disorders. Preliminary results from this questionnaire and the relationship of sleep apnea with obesity in the 2005-2006 dataset have been described elsewhere [30]. Even though both the 2005-2006 and 2007-2008 datasets contain data on the sleep questionnaire, currently the plasma homocysteine levels are available for the 2005-2006 cycle only. Therefore, we included this dataset for our analysis. The NHANES 2005-2006 survey was approved by the NCHS Ethics Review Board, and all participants or parents (for minors) provided written consent. Total plasma homocysteine levels were determined using the fully automated fluorescence polarization immunoassay (Abbott Laboratories). The RBC and plasma folate estimations were conducted using the Quantaphase II folate/vitamin B12 radioassay (Bio-Rad Laboratories, Hercules, CA, USA) using ^125^I and ^57^Co as tracers. Detailed description of the NHANES 2005-2006 survey can be found online at http://www.cdc.gov/nchs/nhanes.htm.

### 2.2. Outcomes and Predictors

Our primary outcome of interest was presence of self-reported sleep apnea. Although the association of plasma homocysteine has been predominantly examined in the context of OSA, the NHANES sleep questionnaire did not explicitly probe into the type of sleep apnea. We therefore used the diagnosed, self-reported sleep apnea as our outcome of interest. Our primary predictors were plasma homocysteine levels and RBC and plasma folate. However, as described previously using this dataset, there were additional variables that were (or could have been) associated with altered risk of sleep apnea. These variables were age, gender, race, country of birth, obesity, hypertension, ever smoking, and ever alcohol use. We examined the potential association of these sociodemographic and clinical variables with the reported diagnosis of sleep apnea and included the significant variables as secondary covariates in multivariate models. For these analyses, we dichotomized plasma homocysteine, RBC folate, and age based on the basis of the receiver operating characteristic (ROC) curves. The optimum cut-off points for these variables were obtained at ≥8.02 *μ*mol/L, ≥279 ng/mL, and ≥47 years, respectively. Education status was dichotomized as high (high school or above) or low (up to and including 11th grade), marital status as married or other, obesity was defined as BMI > 28 Kg/m^2^, and hypertension was defined as an average (mean of three readings) systolic blood pressure > 140 mmHg and/or a diastolic blood pressure > 90 mmHg. Ever smoking was defined as having smoked at least 100 cigarettes in lifetime while ever alcohol use was defined as having had 12 alcoholic drinks in life time.

### 2.3. Statistical Analysis

Descriptive statistics included the means and standard errors (for continuous variables) and proportions (for discrete variables). Statistical significance for difference across study groups was assessed using the Student's *t* test (for continuous variables) or chi-square test (for categorical variables). For binarizing continuous variables, we made use of the ROC and selected the optimum cut-off point by finding the shortest distance from the upper-left corner of the ROC plot. The distance of a point on the ROC from the upper left corner was estimated as $=\sqrt{\left({\left(1-Sn\right)}^{2}+{\left(1-Sp\right)}^{2}\right)}$, where *Sn* is the sensitivity and *Sp* is the specificity of the binarized variable to predict self-reported sleep apnea. We estimated two important effect measures: age-standardized, design-effect-corrected prevalence rates of self-reported sleep apnea in various subgroups and the design-effect-adjusted multivariate association of the above-mentioned predictors with the risk of self-reported sleep apnea.

To determine the potentially independent association of high plasma homocysteine with self-reported sleep apnea, we decomposed the observed total plasma homocysteine levels into age-independent and age-dependent components using the following approach. We fitted a linear regression model HCY = b * age + *c*, where HCY is the plasma homocysteine level, *b* is the regression coefficient, and *c* is constant. Using the results of this model, we generated the two components as HCY_dep_ = b * age and HCY_ind_ = HCY  − HCY_dep_, where the suffixes dep and ind indicate the age-dependent and -independent components, respectively. We then conducted multivariate logistic regression analysis to examine the association of the HCY_ind_ and HCY_dep_ components on the risk of self-reported sleep apnea. For all analyses, we used the svy commands contained in the Stata 12.0 statistical software (Stata Corp, College Station, TX, USA). These commands help account for the survey design effect. The survey was a single-stage, 30-cluster design, and we used the procedure described by the Centers for Disease Control to set the survey data in Stata. To calculate the prevalence rates, we used the svy: mean command with the stdize option while, to conduct the multivariate analyses, we used the svy: estimate command. Statistical significance was evaluated at a type I error rate of 0.05.

## 3. Results

### 3.1. Characteristics of Study Subjects

Plasma homocysteine levels, RBC folate levels, sleep questionnaire responses, and demographic information were available on 4490 subjects in the NHANES 2005-2006 dataset of whom 177 (3.94%) reported past diagnosis of sleep apnea. The distribution of the sociodemographic and clinical variables in subjects with and without reported sleep apnea is shown in Table 2. We found that the subjects with self-reported sleep apnea were on an average over 8 years older than subjects who did not report sleep apnea. Interestingly, the proportion of males, non-Hispanic Whites, and subjects born in the US were ~18% higher than the respective proportions in subjects with self-reported sleep apnea as compared to those without it. Also, a higher proportion of subjects with self-reported apnea were more educated and married. We also observed that the persons with self-reported sleep apnea had a higher body mass index, a higher percentage of ever smokers, and ever alcohol users as compared to those without sleep apnea. There was no significant difference in the economic status of the families as indicated by the poverty income ratio.

### 3.2. Prevalence of Self-Reported Sleep Apnea Based on Plasma Homocysteine and RBC Folate Levels

We observed that the mean plasma homocysteine and mean RBC folate levels were significantly higher in subjects with self-reported sleep apnea (Table 2), but the mean plasma folate levels were comparable in subjects with or without self-reported sleep apnea. Yet, we found that there was a negative correlation of the plasma homocysteine levels with both the RBC and plasma folate levels (*r* = −0.08, *P* < 0.0001 and *r* = −0.03, *P* = 0.0321, resp.).

**Figure 1:**
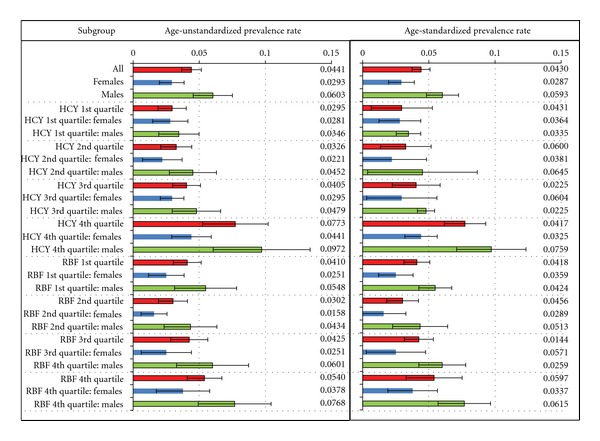
Prevalence of diagnosed, self-reported sleep apnea in the NHANES 2005-2006 dataset based on gender and quartiles of plasma homocysteine and RBC folate. The plot on the left is not standardized for age while the plot on the right shows age-standardized rates. Prevalence rates are shown as horizontal bars, and the estimates are indicated by the value at the right side of the plots. Error bars indicate the 95% confidence interval for the prevalence rates. Prevalence rates are shown for all subjects (red bars), females only (blue bars) and males only (green bars). Range of values for plasma homocysteine and RBC folate represented by their respective quartiles is described in text. HCY: plasma homocysteine; RBF: RBC folate.

Considering the several observed associations of the sociodemographic, clinical, and biochemical variables with self-reported sleep apnea, we first estimated the prevalence of self-reported sleep apnea in various subgroups defined by these variables. We observed (Figure 1) that the overall age-standardized prevalence rate of 4.3% was significantly differential across gender; males had a prevalence rate of ~6% while females had a prevalence of ~2.9%. Thus, we next estimated age-standardized prevalence rates for other subgroups separately for males and females. Since we had observed significant differences in the mean plasma homocysteine and RBC folate (but not plasma folate) across self-reported sleep apnea status, we first categorized these two variables into four groups based on the respective quartiles. The quartiles were generated for all subjects irrespective of the self-reported sleep apnea status. The quartiles for plasma homocysteine were as follows: Q1, <6.23 *μ*mol/L; Q2, 6.23–7.76 *μ*mol/L; Q3, 7.77–9.75 *μ*mol/L; Q4, >9.75 *μ*mol/L. The quartiles for RBC folate were Q1, <207 ng/mL; Q2, 207–268 ng/mL; Q3, 269–354 ng/mL and Q4, ≥355 ng/mL.

We observed that there was a consistent increase in the age-standardized prevalence of self-reported sleep apnea over the four quartiles of plasma homocysteine levels in both males and females albeit the prevalence rates were always lower in females. However, a similar consistent trend was not observed for the quartiles of RBC folate. With the exception of the lowest quartile for RBC folate, the remaining three quartiles demonstrated a consistent increase in the age-standardized prevalence of self-reported sleep apnea. Again, this trend was observed in males as well as females with consistently lower rates in females.

**Figure 2:**
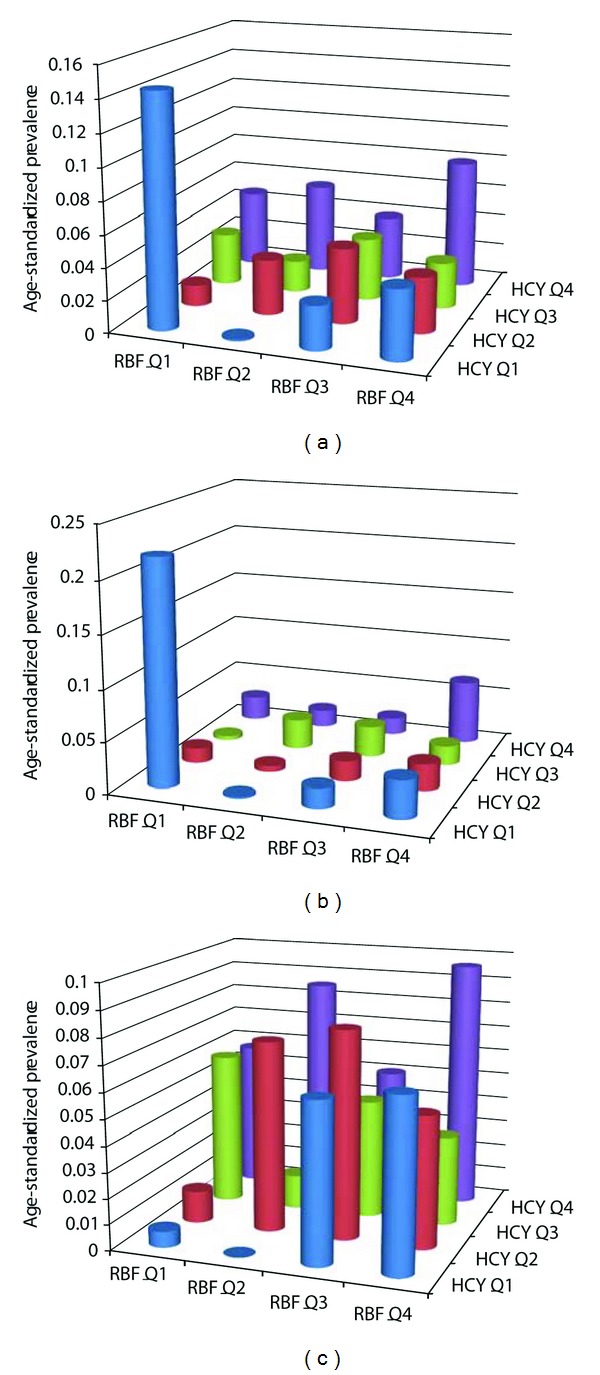
Age-standardized prevalence rates of diagnosed, self-reported sleep apnea based on combinations of quartiles of plasma homocysteine and RBC folate levels. Plots are for all subjects (a), females only (b), and males only (c). HCY: plasma homocysteine; RBF: RBC folate; Q: quartile.

We next estimated the age-standardized prevalence rates for a combination of the plasma homocysteine and RBC folate quartiles (Figure 2). We found some interesting patterns in these analyses. First, females with very low levels of plasma homocysteine as well as RBC folate had a very high prevalence of self-reported sleep apnea. Second, low levels of both plasma homocysteine and RBC folate were associated with a very low prevalence of self-reported sleep apnea in males. Third, highest prevalence of self-reported apnea in the males was found in those who had high level of both plasma homocysteine and RBC folate. Fourth, the mechanistically expected high prevalence of self-reported apnea in the low RBC folate/high plasma homocysteine subgroup was observed in males only but not in females. Together these findings indicated that there existed a complex and surprising combinatorial association of plasma homocysteine and RBC folate levels with self-reported sleep apnea.

### 3.3. Multivariate Association of Plasma Homocysteine and RBC Folate with Self-Reported Sleep Apnea

**Figure 3:**
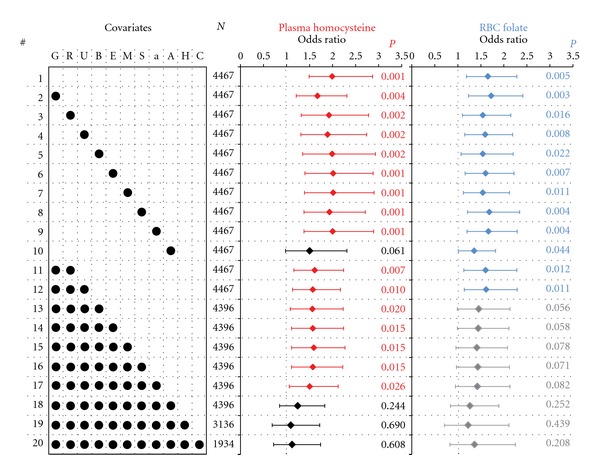
Multivariate association of high plasma homocysteine and RBC folate levels with the risk of self-reported sleep apnea. Results are shown as point (diamonds) and 95% confidence intervals (error bars) for odds ratios estimated through a series of nested logistic regression commands. Twenty logistic regression models (indicated by # on the left) were run with varying combinations of covariates. The covariates included were G: male gender; R: non-Hispanic white race; U: birth in the United States; B: body mass index > 28 Kg/m^2^; E: high education; M: married; S: ever smoker; a: ever alcohol use; A: age > 46 years; H: hypertension; C: cardiovascular disease. Model 1 contained only high plasma homocysteine and high RBC folate as the independent variables. The results from models 19 and 20 cannot be directly compared with the remaining 18 models since the information for hypertension and cardiovascular disease was not available for a large number of subjects (shown under column titled *N*). Statistically significant associations (when the error bars did not straddle unity indicated by dashed vertical lines) are shown in red color for high plasma homocysteine and in blue color for high RBC folate. Statistically nonsignificant associations are shown in black color for plasma homocysteine and gray color for RBC folate. Statistical significance is shown on individual plots as color-coded *P* values.

With the use of a series of 20 nested multivariate logistic regression models, we determined if inclusion of epidemiologically important significant covariates influenced the association of plasma homocysteine and RBC folate with self-reported sleep apnea (Figure 3). When no covariates were included along with the two primary predictors, we found that both were independently associated with the risk of self-reported sleep apnea (Model 1, Figure 3). The addition of other important variables like gender, non-Hispanic White race, birth in the United States, education, marital status, obesity, ever smoking, and ever alcohol use somewhat decreased the strength of the associations of plasma homocysteine and RBC folate with self-reported sleep apnea, however, in most instances, the associations retained their statistical significance. Interestingly however, when age was added to the multivariate model (Models 10 and 18, Figure 3), the significance of both plasma homocysteine and RBC folate reduced drastically. The results of the full model (Model 18 in Figure 3) are shown in detail in Table 3.

It is noteworthy that high BMI, male gender, age > 46 years, alcohol use, high education, and married status continued to demonstrate independent association with self-reported sleep apnea in a multivariate context, but the association of high plasma homocysteine and high RBC folate became statistically insignificant (Table 3). Indeed, all the multivariate models (Models 18, 19, 20) that contained age as a covariate demonstrated a nonsignificant association of plasma homocysteine and folate with self-reported sleep apnea. Since the information of hypertension and cardiovascular disease was not available on large number of study subjects, we added these variables to the full model (Model 18) and observed that the strength as well as significance of the association of plasma homocysteine and RBC folate with self-reported sleep apnea was further diminished (Models 19 and 20).

The results presented thus far indicated a strong potential influence of age on the association of plasma homocysteine with self-reported sleep apnea. Considering the disposition of the survey data that we analyzed, we further refined our analyses. We examined if the strong positive correlation of age with plasma homocysteine levels (*r* = 0.38, *P* < 0.0001) masked the potentially true, independent association of plasma homocysteine with self-reported sleep apnea. We found that the odds ratio associated with a unit increase in the HCY_dep_ levels was 1.51 (95% confidence interval 1.36–1.68), *P* < 0.0001 while that for a unit increase in HCY_ind_ was 1.02 (95% confidence interval 0.99–1.05), *P* = 0.189. These results demonstrated that the observed overall association of the plasma homocysteine levels with the risk of self-reported sleep apnea was indeed due to age.

## 4. Discussion

In this large, nationally representative sample of noninstitutionalized US subjects, we observed that plasma homocysteine is not independently associated with an altered risk of self-reported sleep apnea. Further, we found that age was the most important confounding factor that could have contributed to an apparent association of plasma homocysteine with the risk of self-reported sleep apnea. It has been shown by other investigators in differing contexts [31–34] that plasma homocysteine levels increase with age. It is also very well recognized that the risk of cardiovascular morbidity rises with age as well as plasma homocysteine levels. However, this concomitant elevation of homocysteine with age appears to be associative rather than causal in the context of sleep apnea. As identified by Lavie et al. [22], Svatikova et al. [26] and Winnicki and Palatini [9] sleep apnea and high plasma homocysteine levels can independently and additively increase the risk of subsequent cardiovascular morbidity; however, a potentially causal link between elevated plasma homocysteine and sleep apnea does not seem to be operative from our results.

Interestingly and intriguingly, we observed a positive association of the RBC folate levels with the risk of self-reported sleep apnea in spite of a mild negative correlation between RBC folate and plasma homocysteine levels. Further, despite a strong positive correlation between plasma and RBC folate (*r* = 0.49, *P* < 0.0001; data not shown), we observed that plasma folate levels were not associated with an altered risk of self-reported sleep apnea. These observations have two important implications. First, these findings indicate that initial enthusiasm generated by the possibility of vitamin supplementation aimed at reducing the prevalence of sleep apnea may be an oversimplification of the apnea challenge. For example, even though folate fortification of grains has been attributed with a decrease in homocysteine levels as well as cardiovascular morbidity [35], such a decrease has not been observed in the prevalence of sleep apnea in the United States. Second, the National Pathology Alliance benchmarking guidelines [36] stipulate that RBC and plasma folate can act as surrogates of each other and that no additional insights are gained by screening samples by both these methods. However, our results demonstrate that these methods may not be redundant and that differential insights can be obtained by a careful examination of the results. Thus, the issue deserves a closer investigation.

Although recruiting a large, representative sample, the current study was an observational study, and, therefore, all limitations implicit in such study designs and compounded by the survey sample selection should be considered while interpreting these results. In addition, it should be noted that the NHANES survey methodology did not use established apnea screening questionnaires but rather recorded self-reported sleep apnea. Therefore, there exist possibilities of missed diagnoses and a consequent misclassification bias. Despite these limitations, our results indicate that increased plasma homocysteine levels, augmented cardiovascular morbidity, and enhanced risk and prevalence of self-reported sleep apnea may actually be representing a syndromic constellation of the aging phenomenon. Consequently, more involved and intense efforts to understand the genetic and environmental contribution and interaction need to be undertaken before a mechanistic understanding, and the public health implications thereof can be fully realized.

## Figures and Tables

**Table 1:** Summary of evidence for and against the association of plasma homocysteine with sleep apnea.

No.	Authors [Ref]	Year	Type of study	*N*	Results
1	Chen et al. [17]	2011	Cross-sectional	102	Severity of OSA is associated with increased homocysteine levels in subjects with ischemic heart disease
2	Basoglu et al. [14]	2011	Case control	36 cases, 34 controls	No association between plasma homocysteine and OSA in obese patients
3	Cintra et al. [18]	2011	Matched case control	75 cases, 75 controls	Cysteine but not homocysteine is differentially distributed across OSA and non-OSA patients
4	Wang et al. [27]	2010	Cross-sectional	83 patients with OSA, 52 without OSA	Oxidative stress might induce high plasma homocysteine levels in elderly patients with OSA
5	Cerbo et al. [16]	2010	Case report	1	Early onset homocystinuria is associated with apneic spells
6	Sariman et al. [25]	2010	Cross-sectional	38 cases of OSA	Plasma homocysteine levels correlate with the severity of OSA
7	Yavuz et al. [28]	2008	Cross-sectional	62 patients of OSA, 12 controls	Plasma homocysteine levels are elevated in patients with OSA
8	Ozkan et al. [23]	2008	Cross-sectional	34 OSA patients, 15 controls	Plasma homocysteine levels are raised in patients with severe OSA
9	Ryan et al. [24]	2007	Cross-sectional	80 patients with OSA, 30 controls	Plasma homocysteine levels are not associated with either the risk or severity of OSA
10	Kumor et al. [21]	2006	Cross-sectional	47 patients of OSA, 12 controls	Plasma homocysteine levels are not differentially distributed across patients and controls of OSA
11	Hachul de Campos et al. [19]	2006	Cross-sectional	38 insomniac postmenopausal women	Plasma homocysteine levels are not associated with risk of apnea
12	Can et al. [15]	2006	Cross-sectional	30 OSA patients, 32 controls	Serum homocysteine levels are significantly higher in OSA patients
13	Kokturk et al. [20]	2006	Cross-sectional	72 OSA patients, 42 controls	Serum homocysteine is significantly increased in patients with OSA
14	Svatikova et al. [26]	2004	Case control	22 OSA patients, 20 controls	Plasma homocysteine levels exhibit diurnal variation and are not differentially distributed across patients and controls of OSA
15	Lavie et al. [22]	2001	Case control	237 cases of OSA, 108 controls	Patients with ischemic heart disease and OSA have elevated plasma homocysteine levels

**Table 2:** Sociodemographic, clinical, and relevant biochemical characteristics of the population included in this study (total *n* = 4490).

Characteristic	Sleep apnea (*n* = 177)	No sleep apnea (*n* = 4313)	*P*
Age (yrs); mean(SD)	56.5 (15.2)	48.1 (19.0)	<0.001
Males; *n* (%)	116 (65.5)	2036 (47.2)	<0.001
Race/ethnicity; *n *(%)			<0.001
Mexican American	11 (6.2)	899 (20.8)	
Other Hispanic	2 (1.1)	136 (3.12)	
Non-Hispanic White	120 (67.8)	2144 (49.7)	
Non-Hispanic Black	40 (22.6)	962 (22.3)	
Others	4 (2.3)	172 (4.0)	
Country of birth; *n* (%)			<0.001
United States	168 (94.9)	3345 (77.6)	
Mexico	4 (2.3)	583 (13.5)	
Elsewhere	5 (2.8)	385 (8.9)	
Education; *n* (%)			0.039
Less than 9th grade	10 (5.7)	543 (12.6)	
9–11th grade	20 (11.3)	670 (15.5)	
High school	48 (27.1)	1018 (23.6)	
Some college education	53 (29.9)	1228 (28.5)	
College graduate	46 (26.0)	849 (19.7)	
Refused/do not know	0 (0.0)	5 (0.1)	
Marital status; *n* (%)			0.016
Married	121 (68.4)	2340 (54.3)	
Widowed	11 (6.2)	388 (9.0)	
Divorced	15 (8.5)	412 (9.6)	
Separated	5 (2.8)	130 (3.0)	
Never married	14 (7.9)	675 (15.7)	
Living with partner	11 (6.2)	365 (8.5)	
Refused to answer	0 (0.0)	3 (0.1)	
Poverty income ratio; mean (SD)	2.84 (1.64)	2.68 (1.59)	0.194
Body mass index (Kg/m^2^); mean (SD)	34.90 (0.62)	28.55 (0.10)	<0.001
Ever smoking; *n* (%)	101 (57.06)	2026 (46.97)	0.008
Ever alcohol use; *n* (%)	41 (23.16)	682 (15.81)	0.009
Plasma homocysteine (*μ*mol/L); mean (SD)	9.49 (3.75)	8.44 (4.62)	0.003
RBC folate (ng/mL); mean (SD)	326.7 (180.4)	297.0 (135.4)	0.005
Plasma folate (ng/mL); mean (SD)	14.65 (15.4)	13.8 (9.5)	0.255

**Table 3:** Results from the full multivariate logistic regression analysis (Model 18 in Figure 3) for the outcome of self-reported sleep apnea.

Covariate	OR	95% CI	*P*
High plasma homocysteine	1.24	0.85–1.83	0.244
High RBC folate	1.26	0.84–1.89	0.252
Male gender	2.22	1.45–3.4	0.001
Non-Hispanic White race	1.21	0.74–1.96	0.420
Birth in the United States	1.89	0.93–3.82	0.073
BMI > 28 Kg/m^2^	7.56	4.14–13.79	<0.001
High education	1.71	1.21–2.41	0.005
Married	1.60	1.07–2.39	0.025
Ever smoker	1.28	0.76–2.14	0.334
Ever alcohol use	2.01	1.3–3.11	0.004
Age > 46 years	2.19	1.39–3.44	0.002

OR: odds ratio; CI: confidence interval; *P*: significance value.
